# Supplementary material for: Informing ASR Treatment Practices in a Florida Aquifer through a Human Health Risk Approach
Source: Int J Environ Res Public Health. 2023 Sep 26;20(19):6833. doi: 10.3390/ijerph20196833 (PMC10572346; doi:10.3390/ijerph20196833)
Supplement: Supplementary file 1 [file ijerph-20-06833-s001.zip › ijerph-2533338-supplementary.pdf]

Supplementary Materials

Informing ASR Treatment Practices in a Florida Aquifer through a Human Health Risk Approach

Table S1. Calculations to estimate the DALYs per pathogen or representative pathogen.

Pathogens	Severity Estimates						
		Mild	Moderate	Severe	Fatal	Total DALYs	References
**Pathogenic *E. coli***	Disease Severity	0.06	0.2	0.28	1	2.98×10^{-3}	[42,43]
	Probability	0.94	0.06	0.09	2.00×10^{-4}		
	Duration (years)	0.02	0.03	0.04	3.1*		
	DALYs	8.8×10^{-4}	3.55×10^{-4}	1.12×10^{-3}	6.2×10^{-4}		
P. aeruginosa	Disease Severity	0.067	—	—	—	9.0×10^{-4}	[44,50]
	Probability	1	—	—	—		
	Duration (years)	0.014	—	—	—		
	DALYs	0.0009	—	—	—		
**Adenovirus (adapted for PV1)**	Disease Severity	0.061	0.202	0.281	1	1.56×10^{-3}	[41,45,51]
	Probability	0.922	0.102	0.0498	3.37×10^{-5}		
	Duration (years)	0.0134	0.0195	0.0211	3.1*		
	DALYs	7.54×10^{-4}	4.02×10^{-4}	3.02×10^{-4}	1.04×10^{-4}		
***Cryptosporidium* spp.**	Disease Severity	0.06	0.2	0.28	—	3.22×10^{-3}	[43,49]
	Probability	0.86	0.12	0.02	—		
	Duration (years)	0.01	0.04	0.09	—		
	DALYs	7.19×10^{-4}	1.02×10^{-3}	4.32×10^{-4}	—		

*Calculated as the difference in life expectancy for an adult in Florida (77.1 years of age [41]) and the age of death from diarrheal disease in the United States (74 years [51]).
